# Dr. Trotter's Reply to Mr. Henry

**Published:** 1803-05-01

**Authors:** T. Trotter

**Affiliations:** Newcastle


### *To the Editors of the Medical and Physical Journal.*

GENTLEMEN,

IN your last Number (50) I perceive a letter signed William Henry, dated Manchester, March 13, 1803, in which my name is introduced in a most extraordinary manner. I cannot help thinking such a subject was foreign to the nature of your Journal.

About the time I quitted my station in the fleet, Mr. Complin, chemist, in Bishopsgate-street, gave me a specimen of his preparation of the acetous acid. I thought well of it; and should have been glad to see it introduced into the naval department, and *officially* authorized him to make use of my name, if it could do him a service; a return which I considered justly due to his attention. Mr. Complin, it appears, availed himself of this permission, and my recommendation accompanies his advertisement.

Mr. Henry soon after sent me a bottle of his *Aromatic Vinegar*, and another half full of Mr. Complin's, to show the superiority of his over the other. This specimen of Mr. Complin's was certainly very inferior to what I had seen before. In his letter he informs me, that he could not think I meant to defend the chemical opinions I had given on Mr. Complin's vinegar. I certainly did not, and gave my name solely with a view to serve an industrious man, and to thank him for his civility to public service.

Mr.

Mr. W. Henry *peevishly* complains that I had made no reply to his father's letter. In the introduction to the third vol. of the MED. NAUTICA, which you have kindly inserted into your JOURNAL, I have made an ample *apology* for all applications of this nature on me as an individual. I have been taxed and burthened with correspondence, and found the necessity of giving it up.

I am not conscious of having invaded any of Mr. Henry's rights, as I was totally ignorant of any exclusive priviledge he could claim on the subject. Many applications, similar to Mr. Complin's, have been made to me as physician of the fleet, and I have uniformly acted as a servant of public service: the *duties of office* to the SICK-BED of the brave man have been paramount to all other considerations.

But my chief inducement for replying to Mr. Henry's letter, is to assure your readers that I have had *no fellow-feeling* in transactions of this kind. I *solemnly* declare to the medical world, that the only bribe I ever took was a bag of green peas from a contractor of vegetables, which was carried immediately to a flag officer of the fleet. And my friends here will vouch, that I have not leaped into a splendid carriage and retinue at the expence of my country, after more than twenty years' service.

*Newcastle,*

*April* 5, 1803.

